# Supplementary material for: Identification of photoperiod-induced specific miRNAs in the adrenal glands of Sunite sheep (Ovis aries)
Source: Front Vet Sci. 2022 Jul 22;9:888207. doi: 10.3389/fvets.2022.888207 (PMC9354845; doi:10.3389/fvets.2022.888207)
Supplement: Supplementary file 1 [file Data_Sheet_1.ZIP › Supplementary file/miRDeep2 file.zip!/miRDeep2 file/Know_Structure/LP42A2/oar-mir-495.pdf]

miRBase precursor : oar-mir-495
Total read count : 9968
oar-miR-495-5p read count : 57
oar-miR-495-3p read count : 9871
remaining reads : 40

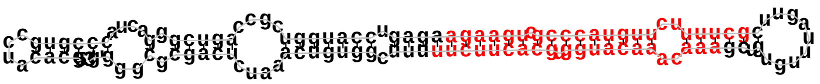

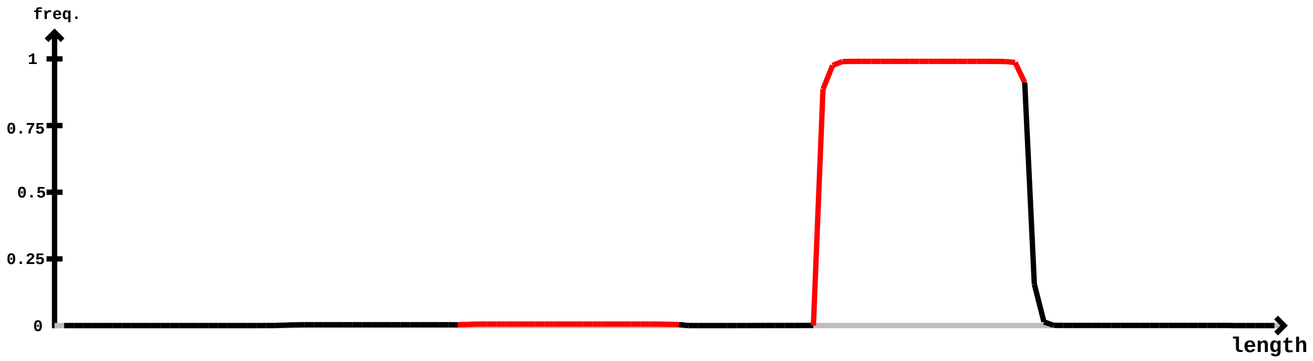


```
                                                                                               oar-miR-495-3p
                                           oar-miR-495-5p
5'- gcacauccgugcccaucagggcugaccgcugguaccugagaagaagucgcccauguucuuuucgcuugauuugugucgaaacaaacauggugcacuucuuuucggugucaaaucucagcgcgggg -3'  exp
    ((((....))))((....(.(((((....(((((((.((((((((((.(((((((((..((((((.........).)))))..))))))).))))))))))).)))))))....))))).)..))  reads  mm
    ....................gcugaccgcugguaccugagaa...................................................................................  1      0
    .....................cugaccgcugguaccugagaa...................................................................................  1      0
    ......................ugaccgcugguaccugag.....................................................................................  1      0
    ......................ugaccgcugguaccugagaa...................................................................................  13     0
    .......................gaccgcugguaccugag.....................................................................................  1      0
    .......................gaccgcugguaccugagaa...................................................................................  10     0
    ........................accgcugguaccugagaa...................................................................................  5      0
    .........................................agaagucgcccauguucuuu................................................................  2      0
    .........................................agaagucgcccauguucuuuu...............................................................  4      0
    .........................................agaagucgcccauguucuuuuc..............................................................  3      0
    .........................................agaagucgcccauguucuuuucg.............................................................  5      0
    ..........................................gaagucgcccauguucuuuu...............................................................  4      0
    ..........................................gaagucgcccauguucuuuuc..............................................................  6      0
    ..........................................gaagucgcccauguucuuuucg.............................................................  33     0
    ......................................................................uugugucgaaacaaacauggugcacuucuu.........................  1      0
    ........................................................................gugucgaaacaaacauggugcacuucu..........................  1      0
    ........................................................................gugucgaaacaaacauggugcacuucuu.........................  1      0
    ............................................................................cgaaacaaacauggugcacuucuu.........................  1      0
    ..............................................................................aaacaaacauggugcacuu............................  6      0
    ..............................................................................aaacaaacauggugcacuuc...........................  31     0
    ..............................................................................aaacaaacauggugcacuucu..........................  666    0
    ..............................................................................aaacaaacauggugcacuucuu.........................  7152   0
    ..............................................................................aaacaaacauggugcacuucuuu........................  943    0
    ..............................................................................aaacaaacauggugcacuucuuuu.......................  36     0
    ...............................................................................aacaaacauggugcacuuc...........................  3      0
    ...............................................................................aacaaaAauggugcacuuc...........................  1      1
    ...............................................................................aacaaacauggugcacuucu..........................  73     0
    ...............................................................................aacaaacauggugcacuucuu.........................  369    0
    ...............................................................................aacaaacauggugcacuucuuu........................  420    0
    ...............................................................................aacaaacauggugcacuucuuuu.......................  20     0
    ................................................................................Gcaaacauggugcacuucu..........................  1      1
    ................................................................................acaaacauggugcacuucu..........................  6      0
    ................................................................................acaaacauggugcacuucuu.........................  23     0
    ................................................................................acaaacauggugcacuucuuu........................  42     0
```

```
                                                                              oar-miR-495-3p
                                         oar-miR-495-5p
gcacauccgugcccaucagggcugaccgcugguaccugagaagaagucgcccauguucuuuucgcuugauuugugucgaaacaaacauggugcacuucuuuuucggugucaaaucucagcgcgggg

................................................................................acaaacauggugcacuucuuuu........................   68   0
................................................................................acaaacauggugcacuucuuuuu.......................   3    0
.................................................................................caaacauggugcacuucuuu.........................   1    0
.................................................................................caaacauggugcacuucuuuu........................   5    0
.................................................................................caaacauggugcacuucuuuuu.......................   1    0
....................................................................................................uuucggugucaaaucucagcg.....   5    0
```
